# Development and Validation of a New Adherence Scale for Antiseizure Medications [ASASM]

**DOI:** 10.3390/jcm13247844

**Published:** 2024-12-23

**Authors:** Sarah A. Alotaibi, Noura A. Alrukban, Layla N. Alanizy, Ahmad Saleh, Bshra A. Alsfouk

**Affiliations:** 1 Department of Pharmaceutical Sciences, College of Pharmacy, Princess Nourah Bint Abdulrahman University, P.O. Box 84428, Riyadh 11671, Saudi Arabia; sarahabdualllah@gmail.com (S.A.A.); ph.noura.alrukban@gmail.com (N.A.A.); 2 Pharmacy Services Administration, King Fahad Medical City, P.O. Box 59046, Riyadh 11525, Saudi Arabia; lalanizy@kfmc.med.sa; 3 Research Center, King Fahad Medical City, P.O. Box 59046, Riyadh 11525, Saudi Arabia; asaleh@kfmc.med.sa

**Keywords:** Adherence Scale for Anti-Seizure Medication(s)-10 items [ASASM-10], antiepileptic drugs, epilepsy, patient-reported compliance, psychometric validation, questionnaire

## Abstract

**Objective:** The objective was to develop and validate a multidimensional scale that measures adherence levels to antiseizure medications and detects patients’ reasons for non-adherence. **Methods:** A new scale was developed, namely the “Adherence Scale for Anti-Seizure Medication(s)-10 items [ASASM-10]”. It consists of ten statements that cover different causes of non-adherence to antiseizure medications. The domain selection was based on a comprehensive literature review. Guidelines for constructing an effective scale were followed to write the statements. Three independent expert judges assessed the content validity of the scale. The reliability of ASASM-10 was tested using three methods: internal consistency measurement (Cronbach’s alpha), Intraclass Correlation Coefficient [ICC] with a 95% Confidence Interval [95% CI], and test–retest reliability. Validity was tested using Principal Component Analysis [PCA] and a correlation coefficient. PCA was applied after measuring sampling adequacy via Kaiser–Meyer–Olkin [KMO] and Bartlett’s sphericity. The Medication Adherence Rating Scale [MARS] was selected as a pre-existing self-report method for validation of this new scale. **Results:** A total of 162 patients completed the study scales (mean ages ± SD: 34.07 ± 10.406 years). The scale demonstrated a good internal consistency with Cronbach’s alpha coefficient of 0.80 and exceeded the required value (i.e., 0.70) for the reliability of new scales. ASASM-10 showed a satisfactory ICC (95% CI) of 0.799 (0.718–0.857), *p*-value < 0.001. The test–retest reliability demonstrated a good correlation of ρ = 0.648, *p*-value < 0.001. The construct validity assessed by PCA retained four components as their eigenvalues exceeded one. The correlation coefficient demonstrated a positive moderate correlation between ASASM-10 and MARS (ρ = 0.283), *p*-value < 0.001. **Conclusions:** The present analyses provided evidence that ASASM-10 is a reliable and valid scale for evaluating patients’ adherence to antiseizure medications. It is the first available scale for assessing medication adherence in patients with epilepsy that can be utilized in clinical practice and research settings.

## 1. Introduction

Medication adherence is defined by the World Health Organization [WHO] as the level to which a patient’s behavior in taking medications corresponds with an agreed treatment plan from healthcare providers [1]. Doubtless, if the patient is non-adherent, either intentionally or unintentionally, to the agreed recommendations, the disease progression and treatment outcomes are adversely affected [2,3]. Medication adherence levels are measured directly or indirectly. Direct detection methods (e.g., drug concentration in blood or urine) are associated with several disadvantages including invasiveness, limited effectiveness, as well as time and effort consumption. On the other hand, indirect methods (e.g., pills count, medication refill, and appointment attendance) have controversial results due to inaccuracy and measurement variability [4,5,6].

Healthcare practice encourages patient-centered care models. Therefore, it is recommended to assess adherence by using a standardized patient-administered questionnaire to determine the patient’s reasons for non-adherence and find a suitable intervention through educational, behavioral, or mixed strategies [6]. There are a number of available questionnaires to measure adherence. The most common ones include the Medication Adherence Rating Scale [MARS] [7], the Drug Attitude Inventory [DAI] [8], and the Medication Adherence Questionnaire [MAQ] [9]. However, there is no validated scale explicitly developed for epilepsy [10]. Epilepsy is a common chronic disease which affects around 50 million persons worldwide [11] and 4/1000 persons in Saudi Arabia [12]. The evidence demonstrates that adherence to anti-seizure medication(s) [ASMs] is suboptimal [6,13,14]. Poor adherence to ASMs is associated with an increased risk of mortality and poorer therapeutic outcomes [2,6,13,15].

Additionally, the available questionnaires are narrowed to specific direct medication-related causes without considering other possible indirect domains that hinder medication adherence in patients with epilepsy such as social reasons and cost of medications [3,16]. In fact, it is essential to be vigilant about all the potential barriers that could interfere with following the health care provider’s recommendations. These common disadvantages of the available tools limit their use and affect the result accuracy. Therefore, there is a clear need for a specific scale that measures adherence to ASMs, covering all the potential reasons for non-adherence to optimize treatment outcomes [17,18].

The aim of this study was to develop a self-administered scale that measures adherence levels to ASMs that can detect all the possible causes for non-adherence. The study also assessed the scale’s reliability and validity.

## 2. Methodology

### 2.1. Development of Adherence Scale for Anti-Seizure Medication(s)—10 Items [ASASM-10]

#### 2.1.1. Selection of Scale Domains

The research team of the present study developed a new scale to measure adherence to ASMs, namely the “Adherence Scale for Anti-Seizure Medication(s)-10 items [ASASM-10]. It consisted of ten statements that cover different causes of non-adherence to ASMs. The main barriers to ASMs adherence were identified by a thorough literature review. In the literature, the main obstacles to adherence include access to ASMs, poor physician–patient relationship, ASMs cost, forgetfulness, frequent medication dosage times, recent uncontrolled seizures, patients’ doubts about their need for ASMs, safety concerns, and epilepsy-related stigma [6,14,15,19,20,21,22,23,24]. Moreover, this novel scale was developed based on the WHO’s five dimensions of medication adherence [1]. As illustrated in Figure S1, adherence factors can be classified into those related to the patient, socio-economic factors, the health care system, the clinical condition, and therapy. Additionally, other rating scales for adherence, such as MARS [7], DAI [8], and MAQ [9], were reviewed and considered for domain selection of this scale.

#### 2.1.2. Scale Development Process

Step 1: Writing scale items

Guidelines for constructing an effective scale [25,26,27] were followed to write the statements of this new scale. The ASASM-10 was built initially in the English language (Appendix S1), then translated into the Arabic language by the research team, who are proficient in both languages. The research team was highly knowledgeable about the topic (i.e., medication adherence in epilepsy) and scale development.

Step 2: Back translation

The ASASM-10 was translated again from Arabic to English by an external translator. The back translator had both pharmaceutical and language professional experience, and was a pharmacy professor at Princess Nourah bint Abdulrahman University [PNU], Saudi Arabia. The back translator was not familiar with the original English scale. Then, the statements in English from the back translation were compared to the original statements. This step was performed to confirm that the Arabic translation was precise and gave similar meaning to the English questionnaire. There were no remarkable differences between the back-translated and original statements.

Step 3: Expert panel review

Three independent expert judges assessed the English and Arabic questionnaires. Two were pharmacy professors at PNU and one was a neurology lecturer at Tanta University, Egypt. The experts judged the scale using a content validity form. All comments were carefully addressed.

Step 4: Pre-test of ASASM-10

The scale was administered to a small number of patients (*n* = 10) with similar characteristics to this study’s target population. This step aimed to identify any problems that may interfere with the patients completing the scale accurately and consistently. No substantial issues were detected.

Step 5: Reliability and validity assessment

After the finalization of the ASASM-10, it was psychometrically tested. The required reliability and validity analyses were conducted. The details, results, and discussion of the scale validation are presented in the following sections.

#### 2.1.3. Scale Scoring and Interpretation

All ten items of the ASASM are dichotomous (agree/disagree), i.e., the patient must respond to each statement with agreement or disagreement based on their feelings and behavior during the past four weeks. For all ten statements of the ASASM, agree responses should be counted as 1 point while disagree responses should be counted as zero points. Therefore, the highest possible score on the scale is ten, and the lowest is zero. Higher scores (i.e., more agree responses on the scale statements) indicate better adherence. In the case of non-adherence, the health care provider can quickly identify the cause(s) because each statement assesses different aspects that can contribute to non-adherence, demonstrated in Table S1. Furthermore, the scale enables the clinician to re-assess the patient’s adherence at every follow-up visit to track adherence, which eventually optimizes treatment goals continuously.

### 2.2. Study Design and Patients

A cross-sectional study was conducted to evaluate the reliability and validity of the ASASM-10. The study included scale self-administration at two different time points and data extraction from patients’ medical records.

The sample size was calculated as 5:1 according to the guidelines for the respondent-to-item ratio (i.e., 50 participants for the 10-item ASASM) [25,28]. Therefore, the sample had to include at least 50 participants for ASASM-10 validation.

Patients were recruited between 1 and 29 July 2021 from outpatient clinics at King Fahad Medical City, Riyadh, Saudi Arabia. A convenient sampling method was used to recruit the eligible patients. Inclusion criteria included patients with a confirmed diagnosis of epilepsy, who were 18 years of age or older, and who had been on ASMs for at least three months. Patients on ASMs for indications other than epilepsy (e.g., post-surgical seizure prophylaxis, brain tumors, migraines, trigeminal neuralgia, bipolar or anxiety disorders) as well as patients who were unable to complete the scale (e.g., illiterate patients or patients with intellectual disability) were excluded from the study.

### 2.3. Procedures and Data Collection

**Figure 1:**
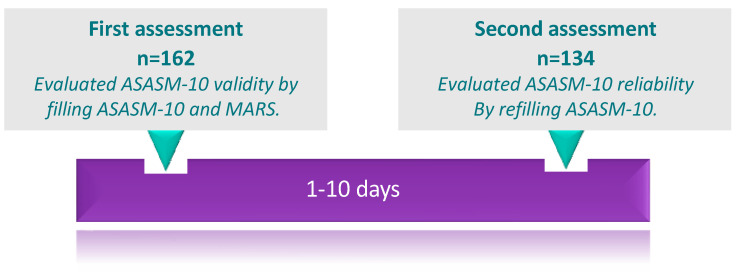
Timeline of scales administration in the study. Abbreviations: ASASM-10, Adherence Scale for Anti-Seizure Medication(s)—10 items; MARS, Medication Adherence Rating Scale.

Eligible patients who agreed to participate in the study completed the study scales. Simultaneously, patients’ demographic and clinical information was collected from their medical records using a pre-designed data collection form. The study procedure for scale administration is demonstrated in Figure 1. To evaluate the scale’s validity, the same group of patients administered both the new scale (ASASM-10) and a pre-existing measure (i.e., MARS) in the first assessment. MARS is a well-known valid measure of medication adherence. MARS is a self-administered measure that includes ten Yes or No questions. Permission to use MARS was taken from the author (Dr Thompson) [7]. The test–retest method was used to assess the scale’s reliability. Therefore, each patient was contacted again at a different time point within 10 days to refill the ASASM-10 for the second assessment.

### 2.4. Statistical Analysis

The Statistical Package for Social Science [SPSS version 25] was utilized to analyze the data. Multi-collinearity for the three scores (ASASM-10 in both first and second assessments and MARS) was tested using the variance inflation factor [VIF]. Consequently, Spearman’s Rho was conducted in correlation tests because the scores for all three scales were not normally distributed. Categorical data were summarized using percentages and frequencies, while continuous data were presented as mean (standard deviation [SD]), median, and range.

#### 2.4.1. Reliability Analyses

The reliability of the ASASM-10 was tested using three methods: the internal consistency measurement (Cronbach’s alpha), the Intraclass Correlation Coefficient [ICC] with a 95% Confidence Interval [95% CI], and test–retest reliability (coefficient of stability). Cronbach’s alpha value of 0.70 or more is considered evidence of internal consistency and reliability [27]. Values of 95% CI of ICC are interpreted as follows: poor reliability at <0.50, moderate reliability at 0.50–0.75, good reliability at 0.75–0.90, and excellent reliability at >0.90 [29]. The coefficient of the stability values of 0–0.20, 0.20–0.40, 0.40–0.60, 0.60–0.80, and 0.80–1.00 are interpreted as a weak, moderate, average, and strong correlation, respectively [30]. The correlation was considered significant at 0.01 level “2-tailed” for the ICC and stability coefficient.

#### 2.4.2. Validity Analyses

The ASASM-10 validity was tested using the Principal Component Analysis [PCA] and a correlation coefficient. PCA was applied after measuring sampling adequacy and significance via Kaiser–Meyer–Olkin [KMO] and Bartlett’s sphericity. KMO ≥ 0.50 provided evidence of an adequate sample size for PCA conduction [31]. A significant *p* value at 0.01 level “2-tailed” for Bartlett’s sphericity test indicated a correlated variable. Eigenvalues describe the variance of each scale item. In a scree plot, those components with eigenvalues of 1 or higher and presented over the sharp decline can be used to identify the number of components principally explaining the data variation [32]. A correlation coefficient was used to evaluate the association between the new and the pre-existing scales used in the first assessment (ASASM-10 scores with MARS scores). The correlation coefficient values of 0–0.20, 0.20–0.40, 0.40–0.60, 0.60–0.80, and 0.80–1.00 are interpreted as a weak, moderate, average, strong, and very strong correlation, respectively [30].

### 2.5. Ethical Considerations and Approvals

The study was approved by the Institutional Review Board [IRB] in King Fahad Medical City, Riyadh, Saudi Arabia (IRB log Number: 21-071). Informed consent was taken from all participants before they started the study. The study purpose and procedures were explained to the patients. The patients were also informed that their participation was optional, did not affect their therapeutic plan, and that they had unconditional rights to withdraw from the study at any time. The patients’ information was confidential.

## 3. Results

### 3.1. Demographic and Clinical Characteristics of the Patients

A total of 162 patients were included in the study. Their ages ranged from 18 to 84 years with a mean (SD) of 34.07 (10.406). Male patients were slightly predominant (*n* = 91, 56.2%). In terms of education, the majority of the patients had undergraduate (*n* = 82, 50.6%) or post-graduate (*n* = 21, 13%) degrees, while only a few patients had primary or secondary school education (*n* = 8, 4.9%). Half of the patients had focal epilepsy (*n* = 81), 34.6% (*n* = 56) had generalised epilepsy, and 15.4% (*n* = 25) had unclassified epilepsy. Epilepsy was controlled (i.e., seizure-free for at least one year) in 42% (*n* = 68) of the patients. Furthermore, ASM regimens were monotherapy in 62 (38.3%) patients and polytherapy in 100 (61.7%) patients. The majority of the patients took their prescribed monotherapy agent in multiple doses per day (*n* = 56, 90.3%) while only 6 (9.7%) patients took their ASM once daily.

Out of 162 participants, 91 (56.2%) had at least one chronic comorbidity, whereas 71 patients (43.8%) were free from any comorbidity. The most common comorbidities were vitamin/mineral deficiency (19.8%), followed by endocrine diseases (17.9%). About 53% of the included patients were on co-medications other than ASMs.

### 3.2. Observed Adherence Scores

The patients were asked to complete the study scales at two different time points that were separated by a period of 1–10 days. The initial assessment (ASASM-10 and MARS) was completed by 162 patients while the second assessment (ASASM-10) was completed by 134 patients. The observed adherence scores of the three assessments are summarised in Table 1.

### 3.3. Reliability of ASASM-10

When the ASASM-10 was applied at two different times, Cronbach’s alpha coefficient was 0.80, which indicated excellent and acceptable evidence of internal consistency for the scale. The ICC (95% CI) for the ASASM-10 from the two-way mixed-effects model was 0.799 (0.718–0.857), *p* value < 0.001. This was interpreted as a statistically significant good reliability of the scale. Furthermore, the test–retest correlation coefficient of the ASASM-10 was ρ = 0.648, *p* value < 0.001, which confirmed a good correlation (0.60 < ρ < 0.80) that was statistically significant.

### 3.4. Validity of ASASM-10

KMO indicated that the sample size was adequate, with a value of 0.538. In addition, Bartlett’s test demonstrated a sufficient result (χ^2^ = 188.820, *p* value < 0.001) to proceed for the PCA test.

**Figure 2:**
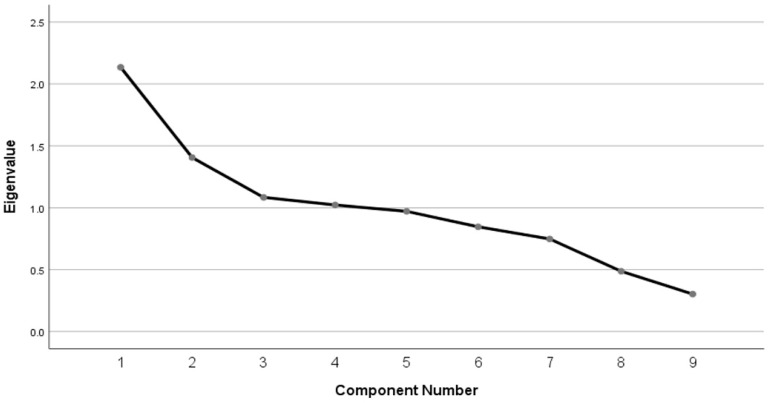
Scree plot of Adherence Scale for Anti-Seizure Medication(s)-10 items [ASASM-10].

Statement 4 of the ASASM-10, which evaluates the impact of medication cost on adherence, was excluded from the analysis because our study was conducted at a governmental hospital in which all medications are free of charge. The eigenvalues of all the components were ≥0.5, except for the 9th and 10th ASASM-10 statements (noted as 8 and 9, respectively). The scree plot indicated four main inflection points, as shown in Figure 2. Four factors were extracted with eigenvalue > 1. Items loadings values are described in Table 2. The first factor seemed to represent the patient’s medication-taking behaviors. The second factor was related to health care system. The third factor represented forgetting to take medication, and the fourth factor may be related to uncontrolled seizures and social stigma.

The correlation coefficient of the ASASM-10 with MARS scores (*n* = 162) demonstrated a positive moderate correlation between the ASASM-10 and MARS (ρ = 0.283), which was statistically significant (*p* value < 0.001).

## 4. Discussion

The primary purpose of this study was to develop a reliable and valid scale that measures adherence to ASMs. The availability of such a scale helps in identifying the adherence level to ASMs and the causes of non-adherence. The present analyses provided evidence that the ASASM-10 was a reliable and valid tool for evaluating patients’ adherence to ASMs. The internal consistency of the ASASM-10 was good with Cronbach’s alpha of 0.80 higher than the required value (i.e., 0.70) for the reliability of new scales [27], and it indicated excellent and acceptable evidence of internal consistency for the scale. Moreover, the ASASM-10 had slightly greater reliability than MARS (Cronbach’s alpha of 0.75) [7]. Among different available reliability estimations, Cronbach’s alpha was selected in this study. It is consistently recommended to use Cronbach’s alpha in the literature and by the best practice guidelines for scale development [25,26,27]. This is due to its ease of interpretation and its ability to provide comparable results across similar studies. It is the most commonly used reliability coefficient in psychometric research, and the reference scale used in this study (MARS) also employed Cronbach’s alpha for its reliability assessment [7].

The test–retest reliability assessment via the correlation coefficient of the ASASM-10 (ρ = 0.648, *p* value < 0.001) proved a statistically significant good correlation, because it was between 0.60 and 0.8 [30]. This correlation coefficient of the ASASM-10 was comparable to that of the MARS findings (ρ = 0.720) [7]. During the scale development process, an expert panel validated the content of the ASASM-10 for both the English and Arabic questionnaires.

Construct validity was assessed by PCA, which retained four components as their eigenvalues exceeded one [32]. The factor analysis of the MARS scale showed three factors [7]. The four factors retained by PCA of the ASASM-10 and its respective items were as follows: Factor 1 primarily represents the patient’s medication-taking behaviors and includes items related to adherence despite seizure absence or side effects, maintaining a consistent medication regimen, and trust in healthcare providers’ knowledge. Factor 2 pertains to healthcare system accessibility, emphasizing the availability of ASMs and ease of accessing healthcare settings. Factor 3 addresses forgetfulness, represented by a single item on consistently remembering to take medication. Factor 4 focuses on uncontrolled seizures and social stigma, including items on seizure control and stigma associated with epilepsy. Both PCA and Exploratory Factor Analysis (EFA) are robust techniques for analyzing construct validity. In this study, PCA was selected. Although PCA is an adequate test for analyzing the construct validity of the scale, performing an EFA with factorial rotation could provide an additional advantage of thoroughly interpreting the variance of factors [33].

Additionally, when the ASASM-10 was compared with the MARS, a moderate significant positive correlation was observed, which confirmed the ASASM-10’s validity for adherence evaluation. However, the moderate correlation observed between the scales may stem from differences in the conditions they assess: the MARS focuses on medication adherence in psychosis, whereas the ASASM-10 evaluates adherence in epilepsy. Furthermore, the ASASM-10 includes unique domains not addressed by the MARS, such as accessibility to healthcare services, physician–patient relationships, medication affordability, social factors, healthcare system efficiency, and stock availability.

The MARS was selected as a pre-existing self-report method for validation of this new scale because it has good validity and reliability; consists of ten Yes or No items which is close to the structure of the ASASM; and integrates both attitudes to taking medication from DAI [8] and medication adherence behaviors MAQ [9].

The ASASM-10 represents the first available validated scale for measuring medication adherence in epilepsy. It has several advantages, including the simplicity in administration and interpretation, and therefore it is a practical tool in both clinical and research settings. Furthermore, this scale is a multidimensional tool that could help to identify and address what individual patient perceives as a barrier to ASM adherence. For example, if the detected patient’s reason for non-adherence is forgetfulness, then a suitable intervention can be a reminder, such as pillbox or alarm [1,24]. Simplification of ASM regimens can be an appropriate strategy for obstacles due to multiple dose frequency [1,6]. Social support can be an effective approach for adherence barriers related to stigma [1,6]. If the non-adherence reason is the patients’ doubts about their need for ASMs, then educational interventions and warning patients about the association between sudden unexpected death in epilepsy (SUDEP) and non-adherence to ASMs can be helpful [34,35].

The content validity of the scale was evaluated by the expert panel review, and criterion validity was conducting by correlating the scores of the ASASM to the pre-existing MARS scores. However, correlating with objective measures, such as plasms drug concentration, was not conducted. Using dichotomous response options (Agree/Disagree) in the scale, ensures simplicity in completion and interpretation. However, this format has limitations, such as preventing neutral responses and failing to capture subtle variations in attitudes or behaviors [26]. Other limitations of this study include the use of a convenience sampling method, which may limit the generalizability of the findings to the broader population. Additionally, reliance on self-report measures introduces the potential for recall bias, as participants may not accurately remember or report their adherence behaviors.

For future studies, we encourage translating and validating the ASASM-10 in other languages and cultures, as well as creating a parent-rated version of the ASASM-10 for measuring adherence to ASMs in children and teenagers. The ASASM-10 shows considerable promise as a reliable and valid tool for assessing adherence to ASMs. The study represents a valuable contribution to the field of epilepsy management. The scale has the potential for wide applicability across clinical and research settings. However, replication studies in diverse populations are needed to confirm its generalizability.

## Figures and Tables

**Table 1:** Observed adherence scores of the three assessments.

	ASASM-10 First Assessment (*n* = 162)	ASASM-10 s Assessment (*n* = 134)	MARS (*n* = 162)
Mean ± SD	8.07 ± 1.678	7.99 ± 1.715	7.80 ± 1.592
Range	3–10	3–10	2–10

The observed scores are out of 10. Abbreviations: ASASM-10, Adherence Scale for Anti-Seizure Medication(s)—10 items; MARS, Medication Adherence Rating Scale; SD, Standard Deviation.

**Table 2:** Component matrix.

Statement	Component			
	1	2	3	4
It is easy for me to visit health care settings (including hospitals and pharmacies) regularly.	0.293	**0.606**	−0.135	−0.356
I trust my health care providers since they have good knowledge about my condition.	**0.452**	0.393	−0.166	−0.333
Whenever I need my anti-seizure medication, it is available in the pharmacy.	0.160	**0.733**	0.037	0.060
I never forget to take my anti-seizure medication.	0.456	−0.077	**0.709**	−0.175
I do not miss a dose of my daily medication regimen due to frequent dosages of my anti-seizure medication or other medications.	**0.610**	−0.057	0.538	0.135
I haven’t had any uncontrolled seizure recently.	0.100	0.366	−0.015	**0.736**
I never stop taking or reducing the dose of my anti-seizure medication even if the seizures have disappeared.	**0.759**	−0.276	−0.329	0.083
I never stop taking or reducing the dose of my anti-seizure medication even if its side effects interfere with daily activities.	**0.749**	−0.344	−0.364	−0.032
I do not feel stigmatized being a patient with epilepsy.	0.303	0.092	−0.064	**0.427**

Extraction method: Principal Component Analysis. Bolded values are the highest value within a row with loading > 0.4. Factor loading with high positive loading indicated strong positive association.

## Data Availability

Noura A. Alrukban and Sarah A. Alotaibi have full access to the study data. The raw data supporting the conclusions of this article will be made available by the authors on request.

## Supplementary Materials

The following supporting information can be downloaded at: https://www.mdpi.com/article/10.3390/jcm13247844/s1, Figure S1: The dimensions of medication adherence according to the World Health Organization (WHO, 2003) [1]. Table S1. Description of the aspect behind each statement of the Adherence Scale for Anti-seizure medication(s)—10 items [ASASM-10]. Appendix S1: Adherence Scale for Anti-seizure Medications-10 (ASASM-10).
