# Supplementary material for: Development and Validation of a New Adherence Scale for Antiseizure Medications [ASASM]
Source: J Clin Med. 2024 Dec 23;13(24):7844. doi: 10.3390/jcm13247844 (PMC11677441; doi:10.3390/jcm13247844)
Supplement: Supplementary file 1 [file jcm-13-07844-s001.zip › jcm-3323937-supplementary.pdf]

Appendix S1

Adherence Scale for Anti-seizure Medications – 10 (ASASM - 10)

Please answer the following questions based on how you have been feeling and doing **during the past four weeks**

Statement	Answer
It is easy for me to visit the health care settings (include hospitals and pharmacies) regularly.	Agree / Disagree
I trust my health care providers since they have good knowledge about my condition.	Agree / Disagree
Whenever I need my anti-seizure medication, it is available in the pharmacy.	Agree / Disagree
The cost of my anti-seizure medication is reasonable.	Agree / Disagree
I never forget taking my anti-seizure medication.	Agree / Disagree
I do not miss a dose of my daily medication regimen due to frequent dosages of my anti-seizure medication or other medications.	Agree / Disagree
I haven’t had any uncontrolled seizure recently.	Agree / Disagree
I never stop taking or reduce the dose of my anti-seizure medication even if the seizures are disappeared.	Agree / Disagree
I never stop taking or reduce the dose of my anti-seizure medication even if its side effects interfere with the daily activities.	Agree / Disagree
I do not feel stigmatized being patient with epilepsy.	Agree / Disagree

<u>The Interpretation:</u>

More agree responses on these statements indicate better adherence.

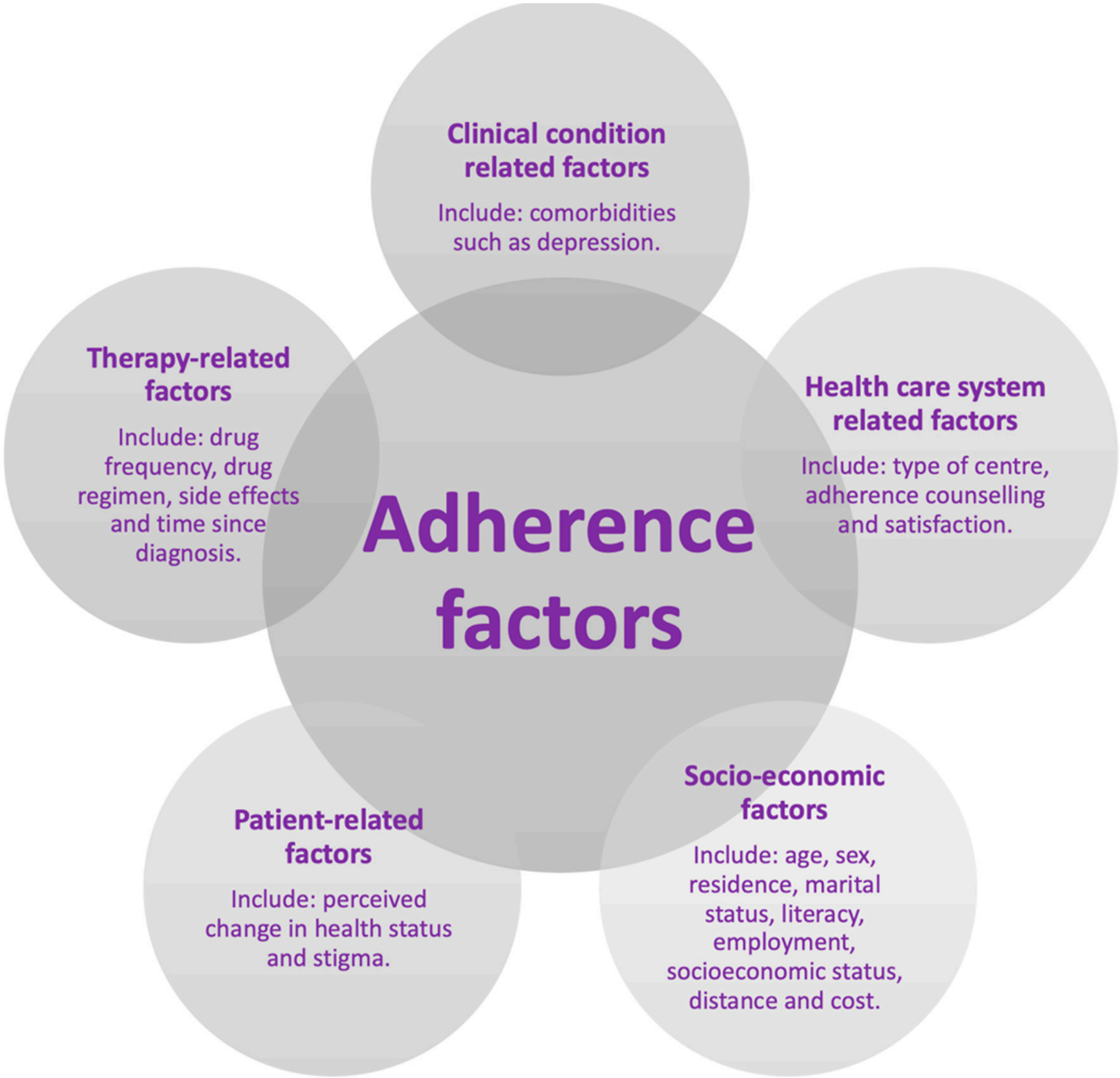

Figure S1. The dimensions of medication adherence according to the World Health Organization (WHO, 2003) [1]

1. World Health Organization. *Adherence to long-term therapies : evidence for action (2003).* https://apps.who.int/iris/handle/10665/42682.

Table S1. Description of the aspect behind each statement of the Adherence Scale for Anti-seizure medication(s) – 10 items [ASASM-10]

Statement	Domain
1. It is easy for me to visit the health care settings (including hospitals and pharmacies) regularly.	A socio-economical aspect related to access to health care settings for follow-up appointments or medication refill such as the distance and cost of transportation.
2. I trust my health care providers since they have good knowledge about my condition.	Relationship between the patient and their health care providers.
3. Whenever I need my anti-seizure medication, it is available in the pharmacy.	Health care system and stock availability.
4. The cost of my anti-seizure medication is reasonable.	Affordability of treatment cost.
5. I never forget taking my anti-seizure medication.	Forgetfulness.
6. I do not miss a dose of my daily medication regimen due to frequent dosages of my anti-seizure medication or other medications.	Complexity of anti-seizure medication(s) regimen and the effect of polypharmacy.
7. I haven't had any uncontrolled seizure recently.	Efficacy of anti-seizure medication(s).
8. I never stop taking or reduce the dose of my anti-seizure medication even if the seizures are disappeared.	Patient understanding for the importance of continue taking anti-seizure medication(s) even when they are seizure-free.
9. I never stop taking or reduce the dose of my anti-seizure medication even if its side effects interfere with the daily activities.	Tolerability of adverse effects of anti-seizure medication(s).
10. I do not feel stigmatized being a patient with epilepsy.	Social and psychological aspects.
